# Structure of the human galanin receptor 2 bound to galanin and Gq reveals the basis of ligand specificity and how binding affects the G-protein interface

**DOI:** 10.1371/journal.pbio.3001714

**Published:** 2022-08-01

**Authors:** Yunseok Heo, Naito Ishimoto, Ye-Eun Jeon, Ji-Hye Yun, Mio Ohki, Yuki Anraku, Mina Sasaki, Shunsuke Kita, Hideo Fukuhara, Tatsuya Ikuta, Kouki Kawakami, Asuka Inoue, Katsumi Maenaka, Jeremy R. H. Tame, Weontae Lee, Sam-Yong Park

**Affiliations:** 1 Structural Biochemistry & Molecular Biophysics Laboratory, Department of Biochemistry, College of Life Science and Biotechnology, Yonsei University, Seoul, Korea; 2 Research Center for Bioconvergence Analysis, Korea Basic Science Institute (KBSI), Chungbuk, Korea; 3 Drug Design Laboratory, Graduate School of Medical Life Science, Yokohama City University, Tsurumi, Yokohama, Japan; 4 PCG-Biotech, Ltd., Seoul, Korea; 5 Faculty of Pharmaceutical Sciences and Global Station for Biosurfaces and Drug Discovery, Hokkaido University, Hokkaido, Japan; 6 Graduate School of Pharmaceutical Sciences, Tohoku University, Sendai, Japan; University of Zurich, SWITZERLAND

## Abstract

Galanin is a neuropeptide expressed in the central and peripheral nervous systems, where it regulates various processes including neuroendocrine release, cognition, and nerve regeneration. Three G-protein coupled receptors (GPCRs) for galanin have been discovered, which is the focus of efforts to treat diseases including Alzheimer’s disease, anxiety, and addiction. To understand the basis of the ligand preferences of the receptors and to assist structure-based drug design, we used cryo-electron microscopy (cryo-EM) to solve the molecular structure of GALR2 bound to galanin and a cognate heterotrimeric G-protein, providing a molecular view of the neuropeptide binding site. Mutant proteins were assayed to help reveal the basis of ligand specificity, and structural comparison between the activated GALR2 and inactive hβ_2_AR was used to relate galanin binding to the movements of transmembrane (TM) helices and the G-protein interface.

## Abstract 2

Galanin is a neuropeptide expressed in the central and peripheral nervous systems, where it regulates various processes including neuroendocrine release, cognition, and nerve regeneration. This cryo-electron microscopy study shows how galanin interacts with one of its three human receptor proteins, GALR2, and reveals the basis of the selectivity of this GPCR for Gq.

## Introduction

Human galanin is a neuropeptide composed of 30 amino acids whose highly conserved N-terminus is linked to biological activities [1]. Its GPCR family receptors (from GALR1 to GALR3) are associated with different disease states and have been used as markers for certain cancer types [2]. Fragments of galanin may have distinct roles from the integral neuropeptide; for example, galanin^2–11^ binds to GALR2 roughly 500 times more tightly than GALR1 [3–5]. Nerve damage induces synthesis of galanin, which appears to show neuroprotective effects mediated by GALR2 [6]. Agonists for GALR2 are believed to have a potential as treatments for a variety of nervous disorders by acting as antidepressants or anticonvulsants as well as having analgesic properties, stimulating nerve growth, and reducing neuronal damage [7–10]. Human GALR2 and human GALR3 show roughly 55% sequence conservation with each other, while human GALR2 shows only 37% sequence identity with human GALR1 [11]. All of them signal via Gi/o and inhibit the adenylyl cyclase, but GALR2 can also activate phospholipase C (PLC) through Gq/11, leading to inositol triphosphate accumulation and a subsequent increase of the concentration of intracellular calcium [12]. In order to understand the signaling pathway of GALR2 and interactions between GALR2 and galanin, we determined the cryo-electron microscopy (cryo-EM) structure of human GALR2 with holo-galanin and associated G-proteins.

## Results and discussion

**Fig 1:**
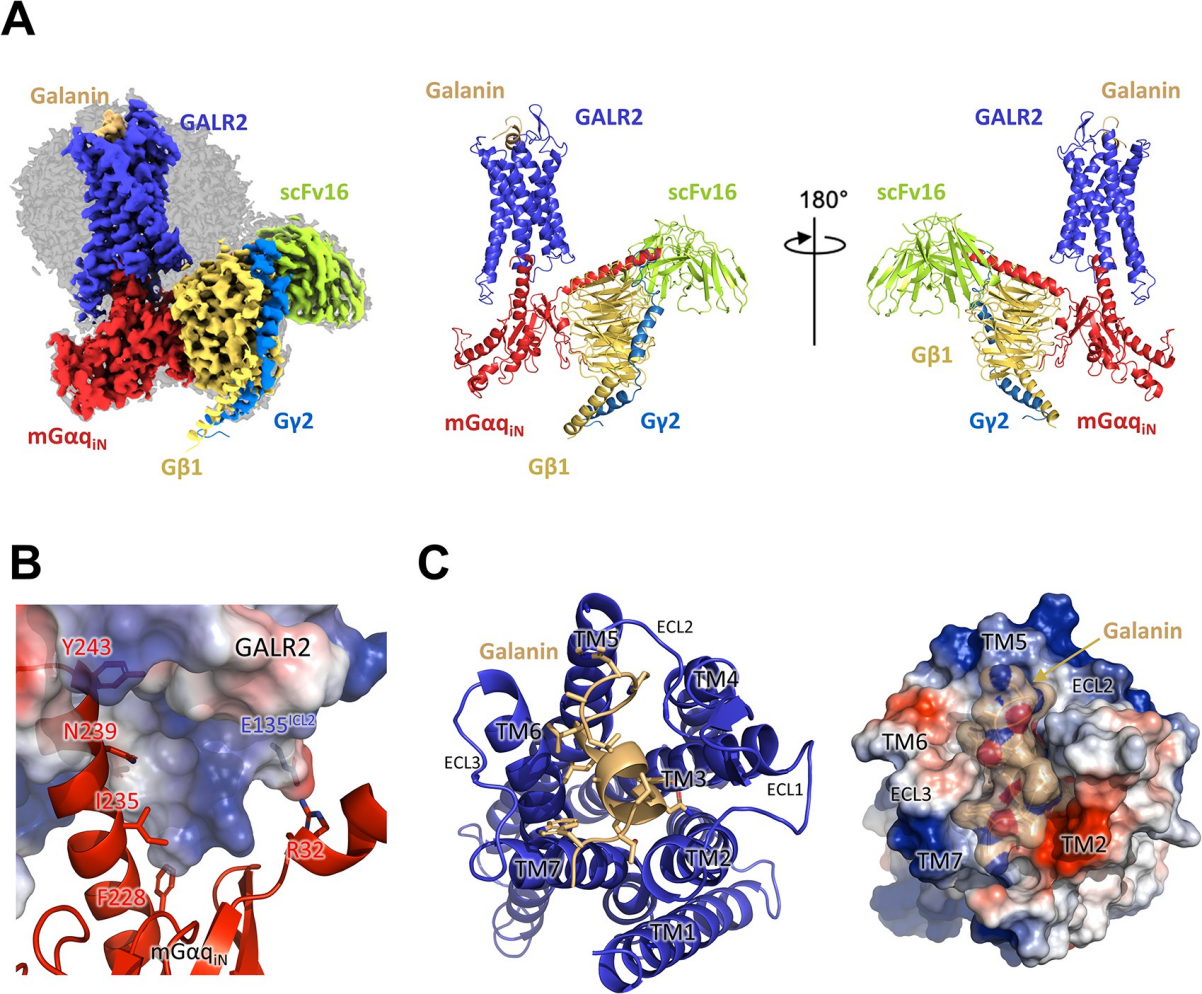
Cryo-EM structure of GALR2 complex with galanin. **(A)** The molecular structure of GALR2 complex is shown both as cryo-EM map and ribbon diagram. GALR2, mGαq_iN_, Gβ1, Gγ2, and scFv16 are colored in blue, red, yellow-orange, marine, and lemon, respectively. The bound galanin peptide is colored in light orange. **(B)** The C-terminal helix of mGαq_iN_ is inserted in the pocket formed by TM5–7 of GALR2 forming hydrophobic and hydrophilic interactions. Arg32 of mGαq_iN_ forms a salt bridge with Glu135^ICL2^ of GALR2. **(C)** The galanin is bound to the pocket formed among the 7 TMs of GALR2 at the extracellular side (left panel). GALR2 and galanin are shown as an electrostatic surface representation (right panel). cryo-EM, cryo-electron microscopy; TM, transmembrane.

To determine the molecular structure of activated GALR2, a complex was purified composed of GALR2, galanin, heterotrimeric mGαq_iN_/Gβ1γ2, and scFv16 (S1 Fig). The sample was plunge-frozen, and micrographs were collected using a 300-kV Titan Krios G4 with a Gatan K3 direct detector in the movie mode. The data were processed with cryoSPARC (v.3.3.1) and were refined with PHENIX (v.1.19.2) (Table 1 and S2A and S2B Fig). The density map was determined to a resolution of 3.11 Å (with FSC cutoff of 0.143). Side chains of GALR2 TM helices are visible for most residues (S2B and S2C Fig). The final model of GALR2 includes all residues from Glu24^1.31^ to Arg300^8.51^, including the intracellular loops (ICLs) and extracellular loops (ECLs). The scFv16 stabilizes the complex by binding to the N-terminal helix of mGαq_iN_ and a surface loop of Gβ1 (Fig 1A).

**Table 1:** Cryo-EM data collection, refinement, and validation statistics.

GALR2-galanin-mGαq_iN_/Gβ1γ2-scFv16	
PDB entry	7XBD
EMDB entry	EMD-33103
**Data collection and processing**	
Magnification	130,000
Microscope	Titan Krios G4
Voltage (kV)	300
Detector	Gatan K3 Summit
Energy filter	Gatan Quantum-LS, 20 eV slit
Electric exposure (e^−^/Å)	53.17
Defocus range (μm)	from −0.8 to −2.0
Collection mode	Standard mode
Pixel size (Å)	0.67
Data Processing Program	CryoSPARC (v.3.3.1)
Movies	7,216
Initial / Final particle images (no.)	2,666,499 / 479,312
Symmetry imposed	C1
Map resolution (Å)	3.11
FSC threshold	0.143
**Refinement**	
Refinement Program	PHENIX (v.1.19.2)
Model resolution (Å)	3.05
FSC threshold	0.143
Model composition	
Non-hydrogen atoms	8,981
Protein residues	1,153
RMS deviations	
Bond length (Å)	0.003
Bond angles (°)	0.531
Validation	
MolProbity score	1.68
Clashscore	6.33
Ramachandran plot	
Favored / Allowed (%)	95.24 / 4.76
Disallowed (%)	0.00
Mask CC	0.72

CC, correlation coefficient; cryo-EM, cryo-electron microscopy; FSC, Fourier shell correlation; RMS, root-mean-square.

**Fig 2:**
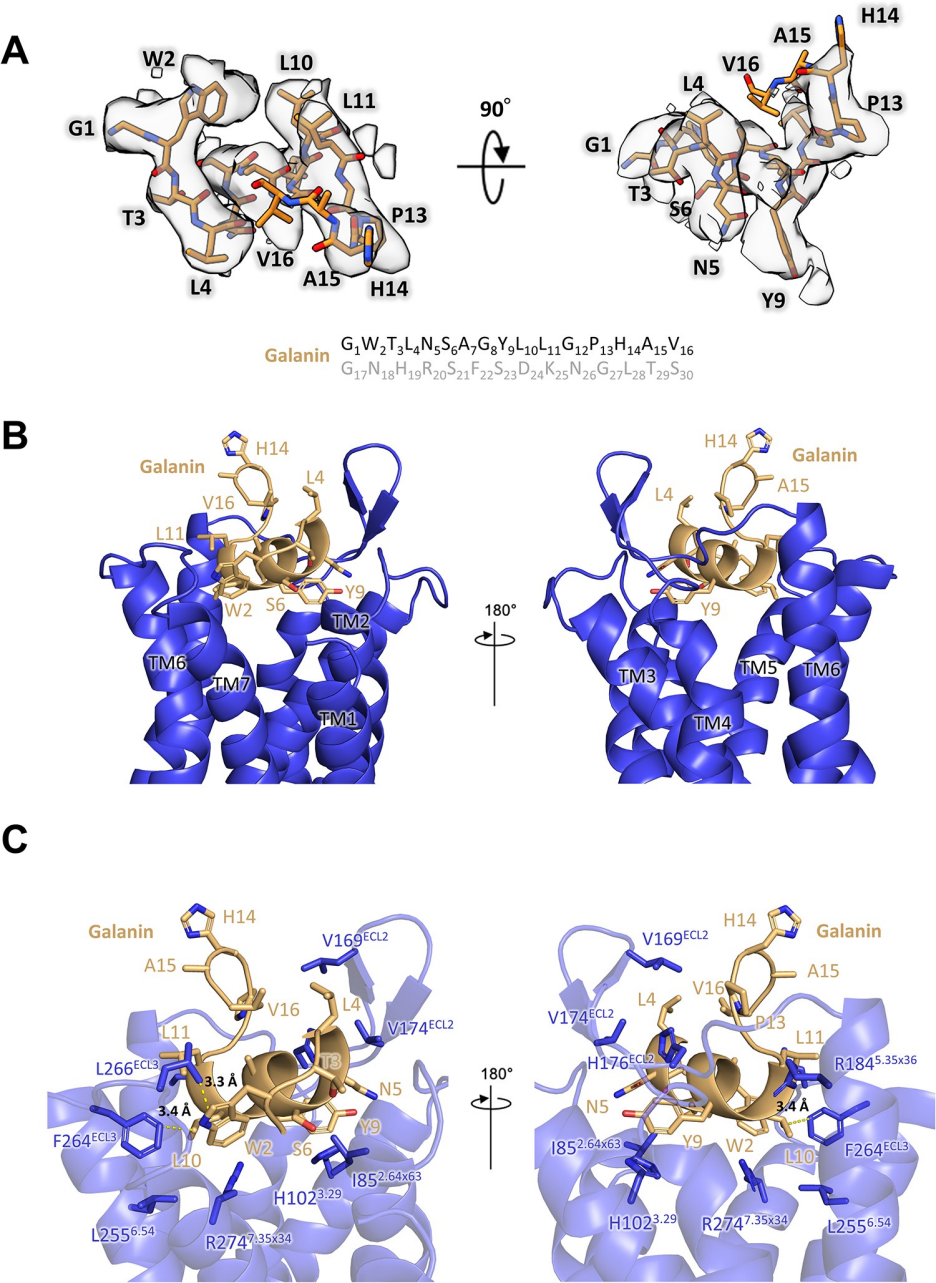
Galanin recognition of GALR2. **(A)** The cryo-EM map near the galanin ligand in 2 different orientations. The sequence of galanin is written below, and the C-terminal 14 amino acids, which are not visible in the map, are colored in gray. **(B)** The ligand binding pocket of GALR2 with galanin is shown in 2 different orientations. GALR2 is shown as a ribbon diagram, and galanin as a ribbon diagram and stick model. **(C)** Detailed interactions between galanin and GALR2. The hydrophobic interactions between Leu10^P^ and Phe264^ECL3^ and Trp2^P^ and Leu266^ECL3^ are indicated with yellow dotted lines. cryo-EM, cryo-electron microscopy.

Activated GPCRs act as nucleotide exchange factors through direct interactions with heterotrimeric G-proteins. The C-terminal helix of mGαq_iN_ fits into a hydrophobic pocket formed by TM 5–7 of GALR2 (Fig 1B). Phe228, Ile235, Asn239, and Tyr243 of mGαq_iN_ form the principal interface with GALR2, and a salt bridge forms between Glu135^ICL2^ of GALR2 and Arg32 of mGαq_iN_ (Fig 1B). The TM helices of GALR2 form a relatively shallow galanin-binding pocket at the extracellular face of the protein (Fig 1C), where the first 16 residues of galanin were modeled into the density map (Fig 2A). The modeled fragment adopts a compact shape with helix-like conformation from Leu4^P^ to Leu11^P^ (Fig 2A). Galanin touches each ECL, and it also forms hydrophobic contacts near the N-termini of TM2 and TM7 (Fig 2B). Galanin has a similar sequence to galanin-like peptide (GALP) and spexin (S3A Fig), which can also act as endogenous ligands and activate GALR2 [13]. Trp2^P^, Thr3^P^, Tyr9^P^, Leu10^P^, and Gly12^P^ of galanin are common to these peptides (S3A Fig). Galanin is well conserved among various species, but the last 15 residues are more variable than the N-terminal region, which is known to interact with GALRs (S3B Fig). Replacing Trp2^P^ of galanin with alanine prevents binding to GALRs [4]. This tryptophan side chain makes a hydrophobic interaction with Leu266^ECL3^ of GALR2 (Fig 2C). The side chain of Tyr9^P^ reaches furthest into the pocket, where it makes interactions with Ile85^2.64×63^, His102^3.29^ and Tyr164^4.64×65^ of the receptor (Fig 2C). Leu10^P^ interacts with Phe264^ECL3^, while Pro13^P^ packs against His176^ECL2^ and Pro177^ECL2^ (Fig 2C). His14^P^, Ala15^P^, and Val16^P^ are completely exposed on the surface of the complex and do not make any interactions with the receptor (Fig 2C). While the majority of the observed galanin residues interact with the receptor to some extent, Trp2^P^, Asn5^P^, and Tyr9^P^ appear to be central to receptor binding, while Leu10^P^ also makes substantial hydrophobic interactions (Fig 2C).

Comparing the sequences of the human galanin receptors (GALR1–GALR3), the majority of the neuropeptide binding site is found to be preserved, but not perfectly. Cys98^3.25^ and Cys175^ECL2^ of GALR2 form a disulfide bridge, and an equivalent bond is presumably also found in the other 2 receptors (S3C Fig). This bond, connecting the N-terminus of TM3 and ECL2 will create a more rigid platform for galanin to pack against. Next to the disulfide bond sits His176^ECL2^, which contacts Asn5^P^, Gly8^P^, and Pro13^P^. This histidine is unique to GALR2 (S3C Fig) and makes a modest contribution to galanin binding; the H176^ECL2^A mutant shows a 5-fold higher EC_50_ value (Table 2). This residue is replaced by tryptophan (Trp188^ECL2^) in GALR1 and valine in GALR3 (S3C Fig), suggesting that the 3 receptors make interactions of different strengths at this position.

**Table 2:** Pharmacological parameters of Gq-signaling activity of GALR2 mutants.

GALR2	Expression level	n	pEC_50_	Stat	EC_50_	Emax	Stat
WT (1:1)		3	8.19 ± 0.11	-	6.4 nM	100	-
WT (1:2.5)		3	7.88 ± 0.08	-	13 nM	92.1 ± 4.2	-
WT (1:5)		3	7.91 ± 0.06	-	12 nM	70.1 ± 8.7	-
WT (1:10)		3	7.83 ± 0.12	-	15 nM	48.3 ± 4.7	-
WT (1:25)		3	7.51 ± 0.11	-	31 nM	27.4 ± 5.1	-
H102^3.29^A	~WT (1:10)	3	6.57 ± 0.02	****	270 nM	17.4 ± 2.4	**
Y164^4.64×65^A	~WT (1:25)	3	NA	NA	NA	NA	NA
Y164^4.64×65^F	~WT (1:2.5)	3	7.77 ± 0.06	ns	17 nM	64.9 ± 2.4	*
V174^ECL2^A	~WT (1:5)	3	6.92 ± 0.09	****	120 nM	72.8 ± 11.3	ns
H176^ECL2^A	~WT (1:1)	3	7.50 ± 0.11	***	32 nM	116.5 ± 3.4	ns
R184^5.35×36^V	~WT (1:5)	3	6.35 ± 0.06	****	450 nM	60.0 ± 6.0	ns
L255^6.54^A	~WT (1:5)	3	7.13 ± 0.06	****	74 nM	64.7 ± 1.6	ns
F264^ECL3^A	~WT (1:1)	3	5.95 ± 0.10	****	1100 nM	90.3 ± 2.3	ns
L266^ECL3^A	~WT (1:1)	3	5.89 ± 0.03	****	1300 nM	90.9 ± 10.5	ns
R274^7.35×34^A	~WT (1:2.5)	3	7.02 ± 0.04	****	96 nM	74.7 ± 5.9	ns

Pharmacological parameters for the Gq-coupling activity analyzed by the NanoBiT-Gq activation assay. Data are presented as mean values ± SEM. EC_50_ was calculated from the mean pEC_50_ value. For Emax calculation, data were normalized to the WT (1:1) GALR2 performed in parallel. NA, parameter not available owing to a lack of ligand response. Statistical analyses were performed using the ordinary one-way ANOVA followed by the Dunnett’s post hoc test with the expression-matched WT response.

ns, *p* > 0.05

*, *p* < 0.05

**, *p* < 0.01

***, *p* < 0.001

****, *p* < 0.0001.

**Fig 3:**
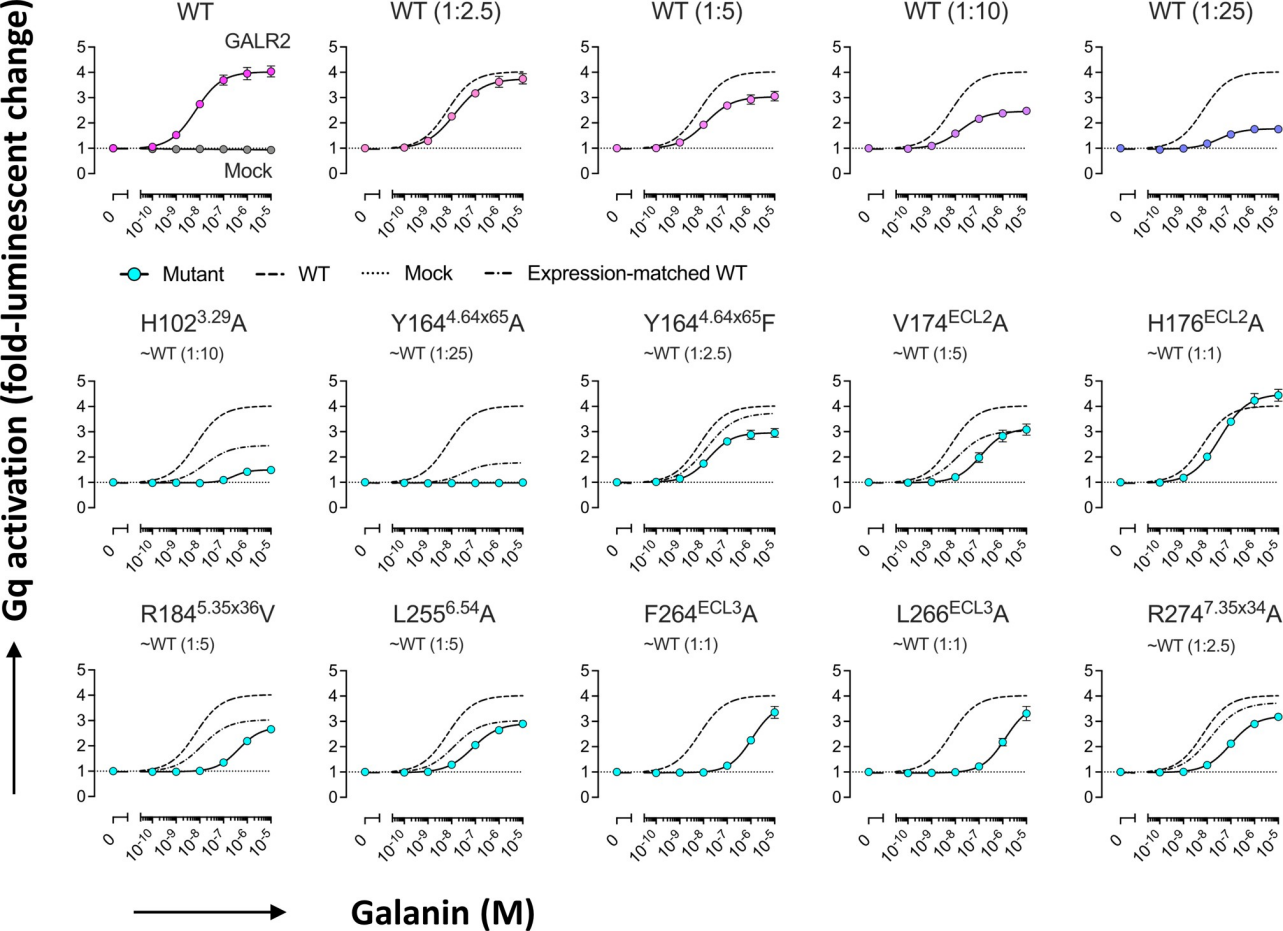
Gq-signaling activity of GALR2 mutants. Galanin-induced Gq-signaling activity of WT GALR2 (titrated plasmid volume) and mutant GALR2 was assessed by the NanoBiT Gq-PLCβ assay. Symbols and error bars indicate mean and SEM, respectively, of 3 independent experiments with each performed in duplicate. The dashed lines, the dotted lines, and long dashed dotted lines represent response curves of WT, mock transfection, and surface expression–matched WT, respectively. Surface expression levels of WT and the mutants both of which contained the N-terminal FLAG-epitope tag were assessed by the flow cytometry using a FLAG-epitope tag antibody (S4A Fig). Note that, in many data points, error bars are smaller than the size of symbols and thus are not visible. The data underlying this figure can be found in S1 Data. WT, wild type.

The GALR2 model was used to design a number of mutants that were assayed for galanin binding, considering the expression level of the mutants (Figs 3, S4A, and S4B). The Y164^4.64×65^F mutant shows only slightly weakened galanin binding, while the Y164^4.64×65^A mutant shows none at all, due to the loss of hydrophobic contact with galanin Tyr9^P^ (Figs 2C and 3). His102^3.29^ is common to all 3 receptors, and H102^3.29^A of GALR2 showed strongly decreased binding affinity to galanin, again emphasizing the importance of Tyr9^P^ for binding (Fig 3). Phe264 and Leu266 in ECL3 contact Leu10^P^ and Trp2^P^, respectively, and replacing either residue with alanine gave roughly 200-fold drops in binding affinity, presumably because ECL3 becomes highly flexible in these mutants, and the same interactions with galanin are lost (Fig 2C and Table 2). Leu255^6.54^ and Arg274^7.35×34^ lie close together and contact Leu10^P^, so that the mutants L255^6.54^A and R274^7.35×34^A both showed 12- to 15-fold drops in affinity for galanin (Fig 2C and Table 2). Mutant R184^5.35×36^V also showed a significant drop in galanin binding, possibly due to its role in stabilizing ECL2 (Fig 3). Although Arg184^5.35×36^ forms a contact with Pro13^P^, the galanin residue is not required for interaction with GALR2. The truncated peptide galanin^2–11^ has been reported to show specificity for GALR2 and GALR3 over GALR1 [3,14]. Duan and colleagues [15] reported that the N-terminal amino group of galanin forms a hydrogen bond with Glu32^1.31^ of GALR1, but no such bond forms with GALR2.

**Fig 4:**
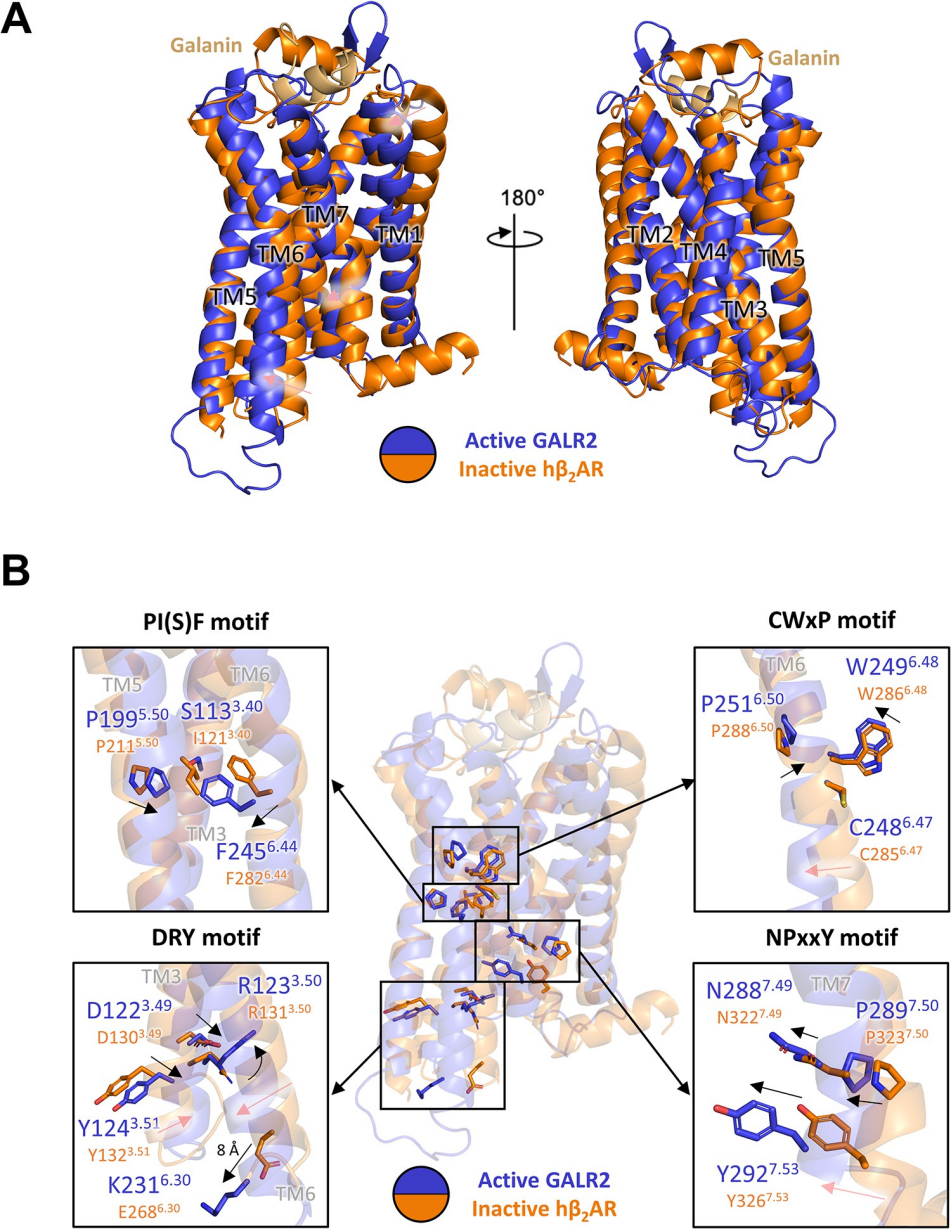
Comparison of the conserved motifs between active GALR2 and inactive hβ_2_AR. **(A)** The overall structures of activated GALR2 and inactive hβ_2_AR in 2 different orientations. GALR2, hβ_2_AR, and galanin are shown as a ribbon diagram. Active GALR2, inactive hβ_2_AR, and galanin are colored in blue, orange, and light orange, respectively. The movements of TM1, TM6, and TM7 are indicated by red arrows. PDB code of the inactive structure of hβ_2_AR is 2RH1. **(B)** The conserved motifs are shown as a stick model. The movements of the residues are indicated by black arrows, and the movements of TM are indicated by red arrows. TM, transmembrane.

The experimental model of activated GALR2 shows notable differences from the inactive model of hβ_2_AR (PDB code 2RH1) [16]. The conformational changes associated with agonist-induced activation of class A GPCRs are well known [17,18], mainly involving a highly conserved “toggle” tryptophan residue (Trp^6.48^) within the CWxP motif of TM6, close to the NPxxY motif of TM7; agonists trigger pronounced movements of TM6 relative to the inactive state, and GALR2 follows the same pattern (Fig 4A and 4B). The DRY and PIF motifs are other motifs common to class A GPCRs, and their conformational changes on activation are shown (Fig 4B). The isoleucine residue of the PIF motif is replaced by serine in GALR2 and GALR3 (S3C Fig). This residue, Ser113^3.40^ of GALR2, shows no significant movement, while Phe245^6.44^ of PI(S)F motif slides against it as TM6 extends toward the cytoplasmic side and rotates, as in the case of other reported GPCR models [17] (Fig 4B). The movement of TM6 is illustrated by the predicted shift of 8 Å in the position of Lys231^6.30^ of the DRY motif (Fig 4B). In common with other class A GPCRs, the shift in TM6 of GALR2 creates a binding site for the cognate G-proteins [17], but unlike the dopamine receptors, the agonist itself (galanin) is considerably distant (>12 Å) from Trp249^6.48^.

Recently, Duan and colleagues published cryo-EM models of GALR1 coupled to G_i_ and GALR2 coupled to G_q_, showing the role of ICL2 in partner selectivity [15]. This model of GALR2 (PDB code 7WQ4) includes the antibody Nb35 used to stabilize the complex, instead of scFv16, and 2 cholesterol molecules are modeled lying against TM6. Therefore, our structure provides an independent view of the complex, showing that the antibodies have little effect on the GPCR itself. Several residues (from 219 to 222) of ICL3 that lie close to G_q_ are not modeled in the structure (PDB code 7WQ4). The RMS deviations between the 273 C^α^ atoms shared by the GALR2 models is 1.16 Å; the largest difference is found around ICL3, which is slightly shifted by indirect effects of Nb35. No notable bonds are formed between ICL3 of the GPCR and G_q_ in our model; Val221^5.72^ comes within 4 Å of Tyr212 of mGαq_iN_. As concluded by the Duan group, the selectivity of GALR2 for G-protein partners is mainly controlled by ICL2 [15]. The protein–protein interface is essentially the same between the model (PDB code 7WQ4) and our model, except that the latter places Glu135^ICL2^ of GALR2 close enough to Arg32 of mGαq_iN_ to form a salt bridge. Although our model includes residues from 1 to 16 of galanin rather than residues from 1 to 13, the ligand overlays closely and makes the same interactions with the protein when compared to each other. Minor changes of rotamer indicate the flexibility of the binding pocket, but there are no significant differences between the ligand interfaces in the 2 GALR2 models.

In conclusion, GALR2 shares the activation mechanism common to class A GPCRs. Our model reveals details of the interactions between GALR2 and galanin, whose N-terminal half adopts a compact form, and this model will assist structure-based drug design selectively targeting one or more galanin receptors to address various human diseases.

## Materials and methods

### Construct design

WT full-length human GALR2 carrying an N-terminal hemagglutinin (HA) signal sequence, FLAG tag, followed by an 8× His tag and HRV 3C protease cleavage site was cloned into the modified pFastBac1 (Invitrogen, Carlsbad, CA, USA) vector. For the GALR2 stability, an additional sequence of A1-L106 encoding thermostabilized apocytochrome b562 RIL (BRIL) with the mutations (M7W, H102I, R106L) was added after the 8× His tag at the N-terminus [19]. The heterotrimeric mGαq_iN_/Gβ1γ2 was designed as previously described in mini-Gαq/Gβ1γ2 [20]. Single chain antibody scFv16 containing GP67 secretion signal sequence was also inserted into pFastBac1 vector [21].

### Expression of GALR2, mGαq_iN_/Gβ1γ2 heterotrimer, and scFv16

GALR2 and mGαq_iN_/Gβ1γ2 were expressed respectively using Bac-to-Bac Baculovirus Expression system (Invitrogen) in *Spodoptera frugiperda* (Sf9) cells using ESF media (Expression Systems, Davis, CA, USA). Using the high titer virus at a multiplicity of infection (MOI) of 3, Sf9 cells density of 2 × 10^6^ cells/mL in 400 mL biomass were infected. The cells were incubated with shaking at 27°C for 72 h and harvested, followed by washing with phosphate-buffered saline (PBS), flash-freezing in liquid nitrogen, and storage at −80°C until further use. The single chain antibody scFv16 was expressed using Bac-to-Bac Expression system in Sf9 cells, and high titer virus was generated. The cells were incubated with shaking at 27°C for 72 h, and secreted scFv16 in the supernatant was separated from the cells by centrifugation.

### Purification of GALR2, mGαq_iN_/Gβ1γ2 heterotrimer, and scFv16

GALR2 frozen pellets were thawed and resuspended at 4°C with the addition of EDTA-free protease inhibitor cocktail (Sigma Aldrich, St. Louis, MO, USA). The cell membranes were obtained by repeated lysis and dounce homogenization using hypotonic buffer containing 10 mM HEPES (pH 7.5), 10 mM MgCl_2_, 20 mM KCl, and protease inhibitors and hypertonic buffer containing 10 mM HEPES (pH 7.5), 10 mM MgCl_2_, 20 mM KCl, 1.0 M NaCl, and protease inhibitors. Washed membrane fractions were resuspended in buffer containing 30 mM HEPES (pH 7.5), 5 mM MgCl_2_, 10 mM KCl, 500 mM NaCl, 200 μM galanin, and protease inhibitors. Full-length galanin was produced by custom peptide order from DGpeptides. Then, membrane fractions were incubated at 25°C for 1 h. Then, solubilized in 1% (w/v) n-dodecyl-β-D-maltopyranoside (DDM) (Anatrace, Maumee, OH, USA), 0.2% (w/v) cholesteryl hemisuccinate (CHS) (Anatrace, Maumee, OH, USA) at 4°C for 3 h. The solubilized solution was isolated by ultracentrifugation at 150,000 × *g* for 1 h and then supernatant was isolated. TALON IMAC (Takara, Sakyo Ward, Kyoto, Japan) resin was added to the supernatant. The mixture was incubated at 4°C, overnight. After incubation, the resin-bound GALR2 was loaded onto a disposable chromatography column (Bio-Rad, Hercules, CA, USA) and the resin was washed with 20 column volumes (CVs) of wash buffer containing 50 mM HEPES (pH 7.5), 500 mM NaCl, 10 mM MgCl_2_, 1% (w/v) DDM, 0.2% (w/v) CHS, 5 mM imidazole, 10% (v/v) glycerol, and 50 μM galanin. Bound proteins were eluted with 10 CVs of elution buffer containing 50 mM HEPES (pH 7.5), 500 mM NaCl, 0.05% (w/v) lauryl maltose neopentyl glycol (LMNG) (Anatrace, Maumee, OH, USA), 0.005% (w/v) CHS, 300 mM imidazole, 10% (v/v) glycerol, and 100 μM galanin. PD-10 desalting column (Cytiva) was used to remove the high concentration of imidazole. GALR2 was then treated overnight at 4°C with HRV 3C protease. Reverse affinity column was used for the further purification of untagged GALR2 with buffer containing 50 mM HEPES (pH 7.5), 500 mM NaCl, 0.05% (w/v) LMNG, 0.005% (w/v) CHS, 10% (v/v) glycerol, and 50 μM galanin. The GALR2 was collected and concentrated, then loaded onto a Superdex 200 Increase 10/300 GL column (Cytiva) with buffer containing 20 mM HEPES (pH 7.5), 100 mM NaCl, 1 mM MgCl_2_, 0.5 mM TCEP, 0.05% (w/v) LMNG, 0.005% (w/v) CHS, and 50 μM galanin via ÄKTA pure system (Cytiva). The fresh GALR2 was used for GALR2-mGαq_iN_/Gβ1γ2 complex formation. mGαq_iN_/Gβ1γ2 frozen pellets were thawed and resuspended at 4°C with the addition of protease inhibitor cocktail. Cells were lysed in lysis buffer containing 20 mM HEPES (pH 7.5), 100 mM NaCl, 1 mM MgCl_2_, 20 mM imidazole, 5 mM β-mercaptoethanol, 100 μM GDP, 1% (v/v) Tergitol-type NP-40 (Sigma), and protease inhibitors. The soluble fraction was isolated by ultracentrifugation at 130,000 × *g* at 4°C for 30 min. The mGαq_iN_/Gβ1γ2 heterotrimer containing soluble fraction was purified using Ni-NTA chromatography and eluted with buffer containing 20 mM HEPES (pH 7.5), 100 mM NaCl, 1 mM MgCl_2_, 300 mM imidazole, 5 mM β-mercaptoethanol, and 10 μM GDP. HRV 3C protease was added and the 6× His tag was cleaved at 4°C for overnight. Reverse affinity column was used for purification of untagged mGαq_iN_/Gβ1γ2. The untagged mGαq_iN_/Gβ1γ2 protein was further purified by size exclusion chromatography (SEC) on a HiLoad 16/600 Superdex 200 column (Cytiva) with following buffer containing 20 mM HEPES (pH 7.5), 100 mM NaCl, 1 mM MgCl_2_, 500 μM TCEP, and 10 μM GDP. The eluted protein was concentrated to 5 mg/mL and stored at −80°C until further use. The supernatant containing scFv16 was loaded onto HisTrap EXCEL column. The column was washed with 10 CVs of wash buffer containing 20 mM HEPES (pH 7.5), 100 mM NaCl, and 50 mM imidazole. The bound protein was eluted using the same buffer supplemented with 500 mM imidazole. After the eluted protein was concentrated, PD-10 desalting column was used to remove the high concentration of imidazole. C-terminal 6× His tag was cleaved by incubation with HRV 3C protease at 4°C for overnight. Reverse affinity column was used for purification of untagged scFv16. The scFv16 was further purified by SEC on a HiLoad 16/600 Superdex 200 column (Cytiva) with following buffer containing 20 mM HEPES (pH 7.5) and 100 mM NaCl. Monomeric fractions were pooled, concentrated, and flash-frozen in liquid nitrogen until further use.

### Formation of GALR2-mGαqiN/Gβ1γ2 complex

For forming GALR2-mGαq_iN_/Gβ1γ2-scFv16 complex, fresh galanin bound GALR2 was mixed with a 1.2 molar excess of mGαq_iN_/Gβ1γ2. The coupling reaction was performed at 25°C for 1 h and followed by addition of 0.2 unit/mL apyrase (New England Biolabs). After additional 1 h at 25°C, lambda phosphatase (New England Biolabs) was added. To make GALR2-mGαq_iN_/Gβ1γ2-scFv16 complex, 1.2 molar excess of scFv16 was added to GALR2-mGαq_iN_/Gβ1γ2 complex and further incubated at 4°C for overnight. The GALR2-mGαq_iN_/Gβ1γ2-scFv16 complex sample in LMNG/CHS was loaded on Superdex 200 Increase 10/300 GL column (Cytiva) equilibrated in buffer containing 20 mM HEPES (pH 7.5), 100 mM NaCl, 1 mM MgCl_2_, 0.5 mM TCEP, 0.001% (w/v) LMNG, 0.0001% (w/v) CHS, and 40 μM galanin. Peak fractions were concentrated to 2.2 mg/mL for electron microscopy studies.

### Cryo-EM grid preparation and data collection

A volume of 3 μl of the sample was applied to a glow-discharged holey carbon grid (Quantifoil R1.2/1.3, Cu, 300 mesh). The grid was blotted with blotting force of 10 for 5 s at 4°C, 100% humidity and flash-frozen into liquid ethane using Vitrobot Mark IV instrument (Thermo Fisher Scientific, Waltham, MA, USA). After the grid was stored in liquid nitrogen, cryo-EM images of the sample were collected on 300 kV Titan Krios G4 (Thermo Fisher Scientific, Waltham, MA, USA) in Hokkaido, Japan. The images were collected by K3-summit camera with 20 eV slit. A total of 7,216 movies were collected by standard mode for 50 frames with total dose of 53.17 e/Å^2^, exposure time of 1.5 s, and dose on camera of 15.967 e^−1^/px/s. Magnification of micrographs were ×130,000, and pixel size was 0.67 Å/pixel. Defocus range was from −0.8 to −2.0 μm. Data were automatically collected using EPU software.

### Cryo-EM data processing

The processing of the collected data were carried out by cryoSPARC (v.3.3.1) [22]. Motion correction was done by Patch motion correction. CTF estimation for micrographs was done by Patch CTF estimation. Micrographs under 5 Å CTF resolution were cut off by Curate Exposures, and 7,040 micrographs were selected. Particles were auto picked by blob picker using only 10 micrographs, and the particles were extracted using binning state (3.35 Å/pixel). Extracting from 10 micrographs, 1,972 particles were picked, and 2D models from these were made. Particles in all micrographs were auto picked by Template picker referenced from 2D models. Approximately 2,666,499 particles were picked from micrographs and extracted using binning state (3.35 Å/pixel). After extracting, all suitable particles were selected and classified by Ab-Initio Reconstruction into 6 classes. Class 2 had the clearest model, and 858,756 particles from the class were selected. After reextracting using the particles in 1.12 Å/pixel (Nyquist Resolution 2.24 Å), 703,468 particles were selected by 2D classification. These particles were classified by Hetero Refinement into 6 classes using Ab-initio model as reference model. After 2 times Hetero Refinement, 3D reconstruction was performed by NU-refinement, and 3.11 Å map was obtained. Finally, the map using DeepEMhancer was used [23].

### Model building and refinement

The first model of GALR2 was used from AlphaFold Protein Structure Database (UniProt:O43603). Model of G-proteins and scFv16 complexes was used from the structure of Cholecystokinin A receptor (CCKAR)-Gq complex (PDB:7EZM) [24]. Models were roughly fitted to the cryo-EM map by COOT at first, and Real-Space Refine in PHENIX was used for further refinement [25]. The model was built manually based on C^α^ and side chain maps using COOT and then refined using Real-Space Refine in PHENIX.

### NanoBiT-based Gq activation assay

Galanin/GALR2-induced Gq activation was measured by the NanoBiT-based Gq-PLCβ association assay [26,27], in which interaction between an activated, GTP-bound Gαq subunit and its effector PLCβ2 is measured by the NanoBiT enzyme complementation system [28]. Plasmid transfection for HEK293A cells (Thermo Fisher Scientific, Waltham, MA, USA) was performed by combining 5 μl (volume is per well in a 6-well culture plate) of polyethylenimine solution (1 mg/mL) and a mixture of plasmids each encoding the Gαq subunit fused with the large fragment (LgBiT) at the amino acid position 123–124 (500 ng), the small fragment (SmBiT)-fused PLCβ2 subunit (500 ng), the untagged Gβ1 and Gγ2 subunits (500 ng each), and the RIC8A chaperone (100 ng), along with a test GALR2 construct (500 ng; containing N-terminal HA-derived signal sequence followed by the FLAG-epitope tag). After an incubation for 1 day, the transfected cells were harvested, pelleted with centrifugation, and suspended in 2 mL of Hank’s balanced saline solution containing 0.01% bovine serum albumin (BSA fatty acid–free grade, SERVA) and 5 mM HEPES (pH 7.4) (assay buffer). The cell suspension was dispensed in a white 96-well plate at a volume of 80 μl per well and mixed with 20 μl of 50 μM coelenterazine (custom-synthesized by Amadis Chemical) diluted in the assay buffer. After 2-h incubation at room temperature, the plate was measured for baseline luminescence (SpectraMax L, Molecular Devices). Thereafter, 20 μl of titrated concentrations of galanin diluted in the assay were manually added, and the plate was positioned for luminescent measurement. Kinetics data points from 5 min to 10 min were averaged and normalized to the initial count and used as a G-protein activation index. The G-protein activation signals were fitted to a 4-parameter sigmoidal concentration-response curve (GraphPad Prism8), and pEC_50_ values (negative logarithmic values of EC_50_ values) and *Span* values (“Top”–“Bottom”) were obtained. For individual experiments, we calculated *E*_*max*_ by normalizing Span to the WT GALR2 and ΔpEC_50_ by subtracting pEC_50_ of the WT GALR2 performed in parallel. We also calculated *E*_*max*_/EC_50_ of GALR2 mutants relative to that of GALR2 WT, a dimensionless parameter known as relative intrinsic activity (RAi) [29] and used its log-transformed value (Log RAi) to denote receptor activity.

### Flow cytometry analysis

Transfection was performed according to the same procedure as described in the “NanoBiT-based Gq activation assay” section. One day after transfection, the cells were collected by adding 200 μl of 0.53 mM EDTA-containing Dulbecco’s PBS (D-PBS), followed by 200 μl of 5 mM HEPES (pH 7.4)-containing Hank’s balanced salt solution. The cell suspension was transferred to a 96-well V-bottom plate in duplicate and fluorescently labeled with an Anti-DDDDK-tag mAb-Alexa Fluor 647 (Clone FLA-1, MBL Life Sciences; 5 μg/mL diluted in 2% goat serum- and 2 mM EDTA-containing D-PBS (blocking buffer)). After washing with D-PBS, the cells were resuspended in 200 μl of 2 mM EDTA-containing-D-PBS and filtered through a 40-μm filter. The fluorescent intensity of single cells was quantified by an EC800 flow cytometer (Sony). The fluorescent signal derived from Alexa Fluor 647 was recorded in an FL3 channel, and the flow cytometry data were analyzed with the FlowJo software (FlowJo). Live cells were gated with a forward scatter (FS-Peak-Lin) cutoff at the 390 setting, with a gain value of 1.7. Values of mean fluorescence intensity (MFI) from approximately 20,000 cells per sample were used for analysis. For each replicate experiment, MFI counts of GALR2 mutant samples were normalized to those of GALR2 WT (100% level) and the mock-transfected samples (0% level), and the resulting values were used to denote surface expression levels of the mutants.

## Supporting information

S1 Fig Purification of recombinant GALR2-galanin-mGαq_iN_/Gβ1γ2-scFv16 complex. After injecting the complex sample into an SEC column, 3 peaks appeared. The complex of GALR2, galanin, heterotrimeric mGαq_iN_/Gβ1γ2, and scFv16 was eluted at the first peak around 10.50 ml. The peak fraction was visualized using an SDS-PAGE. Uncropped gel of this figure can be found in S1 Raw Images.
(TIF)

S2 Fig Cryo-EM processing and TM maps of GALR2. **(A)** The collected data were processed using cryoSPARC. Through 2D and 3D classification, final 479,312 particles were selected for reconstruction. The resolution of GALR2 complex was determined at 3.11 Å. **(B)** FSC curve of GALR2 complex was obtained. Local resolution of GALR2 is shown. The data underlying this figure can be found in S2 Data. **(C)** The atomic models of the 7 TM helices (TM1: 27–51 aa, TM2: 59–87 aa, TM3: 95–128 aa, TM4: 139–160 aa, TM5: 181–214 aa, TM6: 230–261 aa, and TM7: 268–293 aa) are superimposed on the cryo-EM map. aa, amino acid; cryo-EM, cryo-electron microscopy; FSC, Fourier shell correlation; TM, transmembrane.
(TIF)

S3 Fig Sequence alignments of ligands and GALRs. **(A)** The sequences of human galanin, GALP, and spexin are aligned. Totally conserved residues are indicated by red squares, and similarly conserved residues are indicated by yellow squares. The essential region of the ligands for binding to GALR2 is indicated by an orange dotted square. **(B)** The sequences of galanin from various species are aligned. Only the galanin from human is composed of 30 residues. **(C)** The sequences of human GALRs are aligned. The secondary structures of the GALR2 are shown above the sequences. GALP, galanin-like peptide; GALR, galanin receptor.
(TIF)

S4 Fig Surface expression and Gq-signaling activity of GALR2 mutants. **(A)** Flow cytometry analysis of WT and mutant GALR2. N-terminally FLAG-epitope-tagged GALR2 constructs were subjected to the flow cytometry using a FLAG-epitope tag antibody. MFI of the mutants was normalized to WT (1:1) after subtracting that of mock. Symbols and error bars indicate mean and SEM, respectively, of 4 independent experiments (dots) with each performed in duplicate. **(B)** Gq-activity parameters of WT and mutant GALR2. For the individual NanoBiT Gq-PLCβ experiments (Fig 3), RAi of the mutant to that of WT was calculated from Emax and EC50 values, and its logarithm-transformed value (Log RAi) was used to represent Gq-activity parameter. The parameter of Y164A was not available due to the undetectable Gq activity of the mutant. Colors in the mutant bars indicate an expression level matching to that of titrated WT. Bars and error bars represent mean and SEM of 3 independent experiments (dots). NA, parameter not available because of lack of the ligand response. Statistical analyses were performed using the ordinary one-way ANOVA followed by the Dunnett’s post hoc test with the expression-matched (colored) WT response. ns, p > 0.05; ***, *p* < 0.001; ****, *p* < 0.0001. The data underlying this figure can be found in S3 Data. MFI, mean fluorescence intensity; RAi, relative intrinsic activity; WT, wild-type.
(TIF)

S1 Data The numerical values underlying Fig 3. (XLSX)

S2 Data The numerical values of FSC curve in S2B Fig. (XLSX)

S3 Data The numerical values underlying S4A and S4B Fig. (XLSX)

S1 Raw Images Raw gel image of S1 Fig. The injected and eluted SEC samples were analyzed using SDS-PAGE. The red boxes were used to indicate the cropped parts used in S1 Fig.
(TIF)

## Abbreviations:

BRIL: apocytochrome b562 RIL
BSA: bovine serum albumin
CC: correlation coefficient
CHS: cholesteryl hemisuccinate
cryo-EM: cryo-electron microscopy
CV: column volume
DDM: n-dodecyl-β-D-maltopyranoside
D-PBS: Dulbecco’s PBS
ECL: extracellular loop
FSC: Fourier shell correlation
GALP: galanin-like peptide
GALR: galanin receptor
GPCR: G-protein coupled receptor
HA: hemagglutinin
ICL: intracellular loop
LMNG: lauryl maltose neopentyl glycol
MFI: mean fluorescence intensity
MOI: multiplicity of infection
PBS: phosphate-buffered saline
PLC: phospholipase C
RAi: relative intrinsic activity
RMS: root-mean-square
SEC: size exclusion chromatography
Sf9: *Spodoptera frugiperda*
TM: transmembrane
WT: wild-type

## References

[1] Habert-Ortoli E, Amiranoff B, Loquet I, Laburthe M, Mayaux JF. Molecular cloning of a functional human galanin receptor. Proc Natl Acad Sci U S A. 1994 Oct 11;91(21):9780–9783. doi: 10.1073/pnas.91.21.9780 7524088 PMC44900

[2] Šípková J, Kramáriková I, Hynie S, Klenerová V. The galanin and galanin receptor subtypes, its regulatory role in the biological and pathological functions. Physiol Res. 2017 Nov 24;66(5):729–740. doi: 10.33549/physiolres.933576 28730831

[3] Webling KEB, Runesson J, Bartfai T, Langel U. Galanin Receptors and Ligands. Front Endocrinol (Lausanne). 2012 Dec 7;3:146. doi: 10.3389/fendo.2012.00146 23233848 PMC3516677

[4] Land T, Langel U, Löw M, Berthold M, Undén A, Bartfai T. Linear and cyclic N-terminal galanin fragments and analogs as ligands at the hypothalamic galanin receptor. Int J Pept Protein Res. 1991 Sep;38(3):267–272. doi: 10.1111/j.1399-3011.1991.tb01438.x 1722197

[5] Wang S, He C, Hashemi T, Bayne M. Cloning and expressional characterization of a novel galanin receptor. Identification of different pharmacophores within galanin for the three galanin receptor subtypes. J Biol Chem. 1997 Dec 19;272(51):31949–31952. doi: 10.1074/jbc.272.51.31949 9405385

[6] Elliott-Hunt CR, Pope RJP, Vanderplank P, Wynick D. Activation of the galanin receptor 2 (GalR2) protects the hippocampus from neuronal damage. J Neurochem. 2007 Feb;100(3):780–789. doi: 10.1111/j.1471-4159.2006.04239.x 17263796 PMC2705497

[7] Kuteeva E, Hökfelt T, Wardi T, Ogren SO. Galanin, galanin receptor subtypes and depression-like behaviour. Cell Mol Life Sci. 2008 Jun;65(12):1854–1863. doi: 10.1007/s00018-008-8160-9 18500640 PMC11131886

[8] Demsie DG, Altaye BM, Weldekidan E, Gebremedhin H, Alema NM, Tefera MM, et al. Galanin Receptors as Drug Target for Novel Antidepressants: Review. Biologics. 2020 Apr 21;14:37–45. doi: 10.2147/BTT.S240715 32368008 PMC7183331

[9] Lu X, Ross B, Sanchez-Alavez M, Zorrilla EP, Bartfai T. Phenotypic analysis of GALR2 knockout mice in anxiety- and depression-related behavioral tests. Neuropeptides. 2008 Aug;42(4):387–397. doi: 10.1016/j.npep.2008.04.009 18554714 PMC3399724

[10] Holmes A, Picciotto MR. Galanin: a novel therapeutic target for depression, anxiety disorders and drug addiction? CNS Neurol Disord Drug Targets. 2006 Apr;5(2):225–232. doi: 10.2174/187152706776359600 16611095

[11] Jurkowski W, Yazdi S, Elofsson A. Ligand binding properties of human galanin receptors. Mol Membr Biol. 2013 Mar;30(2):206–216. doi: 10.3109/09687688.2012.750384 23237663

[12] Wittau N, Grosse R, Kalkbrenner F, Gohla A, Schultz G, Gudermann T. The galanin receptor type 2 initiates multiple signaling pathways in small cell lung cancer cells by coupling to G(q), G(i) and G(12) proteins. Oncogene. 2000 Aug 31;19(37):4199–4209. doi: 10.1038/sj.onc.1203777 10980593

[13] Mills EG, Izzi-Engbeaya C, Abbara A, Comninos AN, Dhillo WS. Functions of galanin, spexin and kisspeptin in metabolism, mood and behaviour. Nat Rev Endocrinol. 2021 Feb;17(2):97–113. doi: 10.1038/s41574-020-00438-1 33273729

[14] Lu X, Lundström L, Bartfai T. Galanin (2–11) binds to GalR3 in transfected cell lines: limitations for pharmacological definition of receptor subtypes. Neuropeptides. 2005 Jun;39(3):165–167. doi: 10.1016/j.npep.2004.12.013 15944007

[15] Duan J, Shen DD, Zhao T, Guo S, He X, Yin W, et al. Molecular basis for allosteric agonism and G protein subtype selectivity of galanin receptors. Nat Commun. 2022 Mar 15;13:1364. doi: 10.1038/s41467-022-29072-3 35292680 PMC8924211

[16] Cherezov V, Rosenbaum DM, Hanson MA, Rasmussen SGF, Thian FS, Kobilka TS, et al. High-resolution crystal structure of an engineered human beta2-adrenergic G protein-coupled receptor. Science. 2007 Nov 23;318(5854):1258–1265. doi: 10.1126/science.1150577 17962520 PMC2583103

[17] Xu P, Huang S, Mao C, Krumm BE, Zhou XE, Tan Y, et al. Structures of the human dopamine D3 receptor-Gi complexes. Mol Cell. 2021 Mar 18;81(6):1147–1159. doi: 10.1016/j.molcel.2021.01.003 33548201

[18] Koehl A, Hu H, Maeda S, Zhang Y, Qu Q, Paggi JM, et al. Structure of the μ-opioid receptor-Gi protein complex. Nature. 2018 Jun;558(7711):547–552. doi: 10.1038/s41586-018-0219-7 29899455 PMC6317904

[19] Chun E, Thompson AA, Liu W, Roth CB, Griffith MT, Katritch V, et al. Fusion partner toolchest for the stabilization and crystallization of G protein-coupled receptors. Structure. 2012 Jun 6;20(6):967–976. doi: 10.1016/j.str.2012.04.010 22681902 PMC3375611

[20] Kim K, Che T, Panova O, DiBerto JF, Lyu J, Krumm BE, et al. Structure of a Hallucinogen-Activated Gq-Coupled 5-HT2A Serotonin Receptor. Cell. 2020 Sep 17;182(6):1574–1588. doi: 10.1016/j.cell.2020.08.024 32946782 PMC7593816

[21] Heo Y, Yoon E, Jeon YE, Yun JH, Ishimoto N, Woo H, et al. Cryo-EM structure of the human somatostatin receptor 2 complex with its agonist somatostatin delineates the ligand-binding specificity. Elife. 2022 Apr 21;11:e76823. doi: 10.7554/eLife.76823 35446253 PMC9054131

[22] Punjani A, Rubinstein JL, Fleet DJ, Brubaker MA. cryoSPARC: Algorithms for rapid unsupervised cryo-EM structure determination. Nat Methods. 2017 Mar;14(3):290–296. doi: 10.1038/nmeth.4169 28165473

[23] Sanchez-Garcia R, Gomez-Blanco J, Cuervo A, Carazo JM, Sorzano COS, Vargas J. DeepEMhancer: a deep learning solution for cryo-EM volume post-processing. Commun Biol. 2021 Jul;4(1):874. doi: 10.1038/s42003-021-02399-1 34267316 PMC8282847

[24] Liu Q, Yang D, Zhuang Y, Croll TI, Cai X, Dai A, et al. Ligand recognition and G-protein coupling selectivity of cholecystokinin A receptor. Nat Chem Biol. 2021 Dec;17(12):1238–1244. doi: 10.1038/s41589-021-00841-3 34556862 PMC8604728

[25] Afonine PV, Poon BK, Read RJ, Sobolev OV, Terwilliger TC, Urzhumtsev A, et al. Real-space refinement in PHENIX for cryo-EM and crystallography. Acta Crystallogr D Struct Biol. 2018 Jun 1;74(Pt 6):531–544. doi: 10.1107/S2059798318006551 29872004 PMC6096492

[26] Pfeil EM, Brands J, Merten N, Vögtle T, Vescovo M, Rick U, et al. Heterotrimeric G Protein Subunit Gαq Is a Master Switch for Gβγ-Mediated Calcium Mobilization by Gi-Coupled GPCRs. Mol Cell. 2020 Dec 17;80(6):940–954. doi: 10.1016/j.molcel.2020.10.027 33202251

[27] Littmann T, Ozawa T, Hoffmann C, Buschauer A, Bernhardt G. A split luciferase-based probe for quantitative proximal determination of Gαq signalling in live cells. Sci Rep. 2018 Nov 21; 8:17179. doi: 10.1038/s41598-018-35615-w 30464299 PMC6249299

[28] Dixon AS, Schwinn MK, Hall MP, Zimmerman K, Otto P, Lubben TH, et al. NanoLuc Complementation Reporter Optimized for Accurate Measurement of Protein Interactions in Cells. ACS Chem Biol. 2016 Feb 19;11(2):400–408. doi: 10.1021/acschembio.5b00753 26569370

[29] Figueroa KW, Griffin MT, Ehlert FJ. Selectivity of agonists for the active state of M1 to M4 muscarinic receptor subtypes. J Pharmacol Exp Ther. 2009 Jan;328(1):331–342. doi: 10.1124/jpet.108.145219 18824613 PMC2644050

