# Supplementary figures and images for: Structure of the human galanin receptor 2 bound to galanin and Gq reveals the basis of ligand specificity and how binding affects the G-protein interface
Source: PLoS Biol. 2022 Aug 1;20(8):e3001714. doi: 10.1371/journal.pbio.3001714 (PMC9371267; doi:10.1371/journal.pbio.3001714)

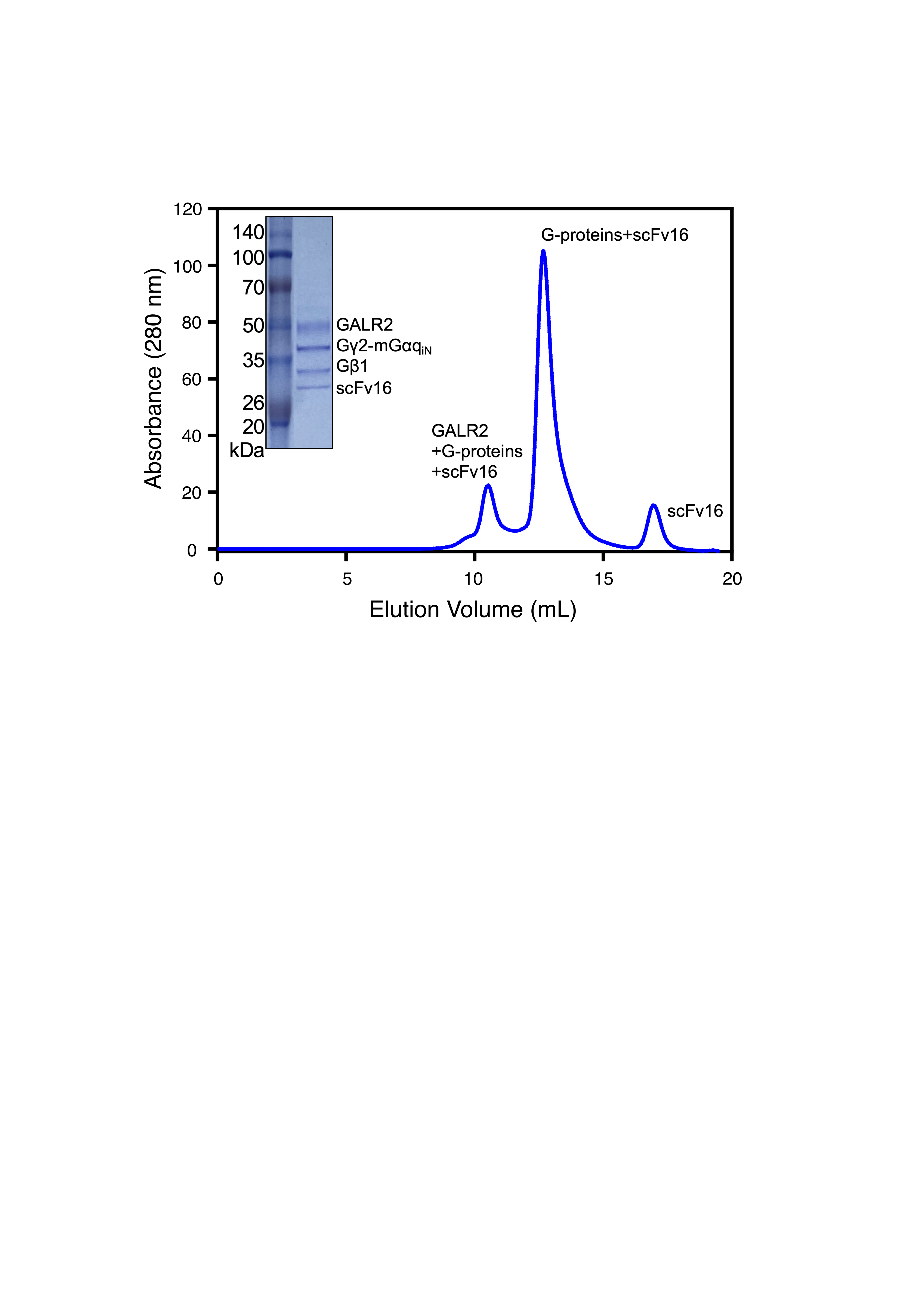

Supplement: S1 Fig — After injecting the complex sample into an SEC column, 3 peaks appeared. The complex of GALR2, galanin, heterotrimeric mGαqiN/Gβ1γ2, and scFv16 was eluted at the first peak around 10.50 ml. The peak fraction was visualized using an SDS-PAGE. Uncropped gel of this figure can be found in S1 Raw Images. (TIF) [file pbio.3001714.s001.TIF]

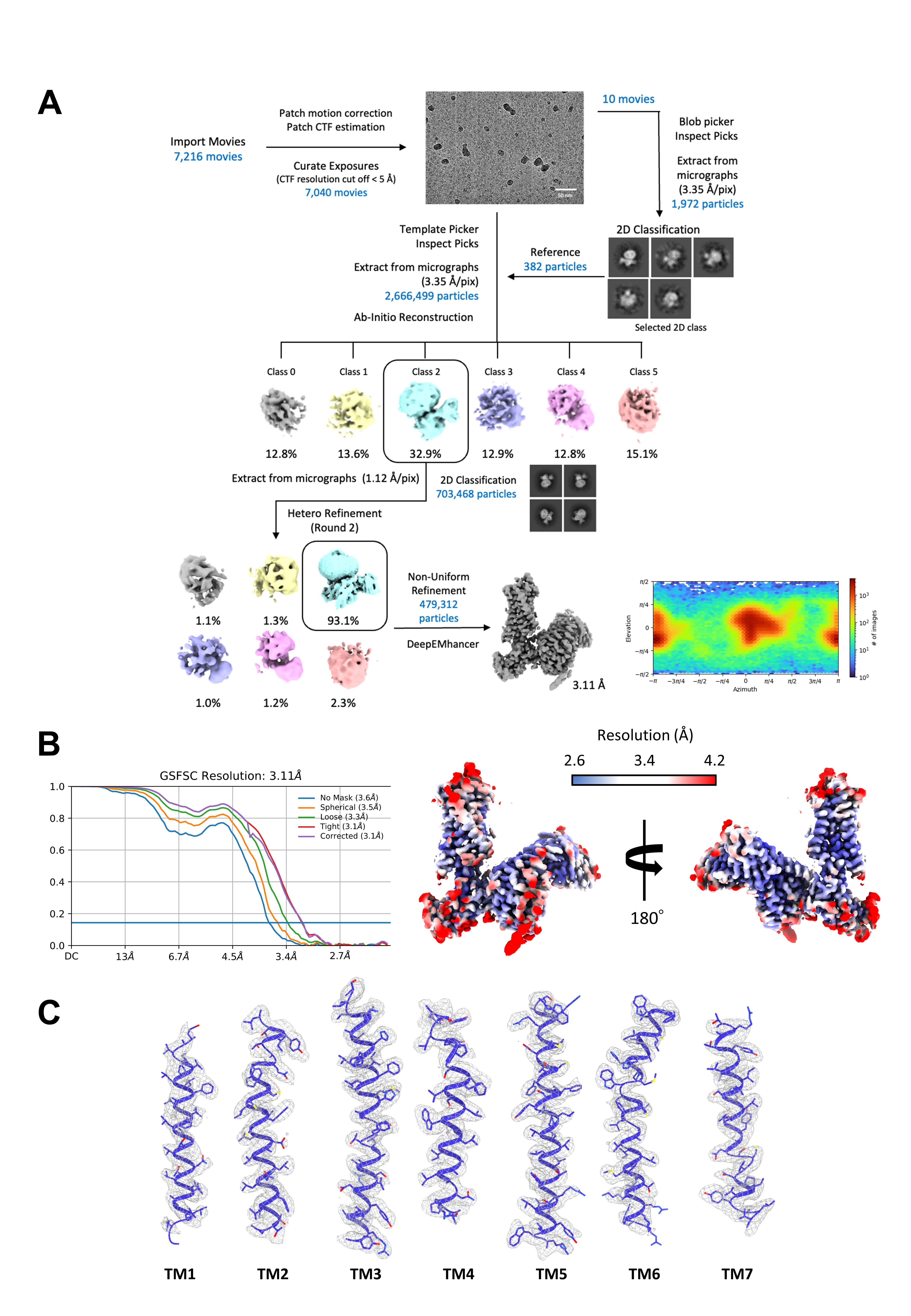

Supplement: S2 Fig — (A) The collected data were processed using cryoSPARC. Through 2D and 3D classification, final 479,312 particles were selected for reconstruction. The resolution of GALR2 complex was determined at 3.11 Å. (B) FSC curve of GALR2 complex was obtained. Local resolution of GALR2 is shown. The data underlying this figure can be found in S2 Data. (C) The atomic models of the 7 TM helices (TM1: 27–51 aa, TM2: 59–87 aa, TM3: 95–128 aa, TM4: 139–160 aa, TM5: 181–214 aa, TM6: 230–261 aa, and TM7: 268–293 aa) are superimposed on the cryo-EM map. aa, amino acid; cryo-EM, cryo-electron microscopy; FSC, Fourier shell correlation; TM, transmembrane. (TIF) [file pbio.3001714.s002.tif]

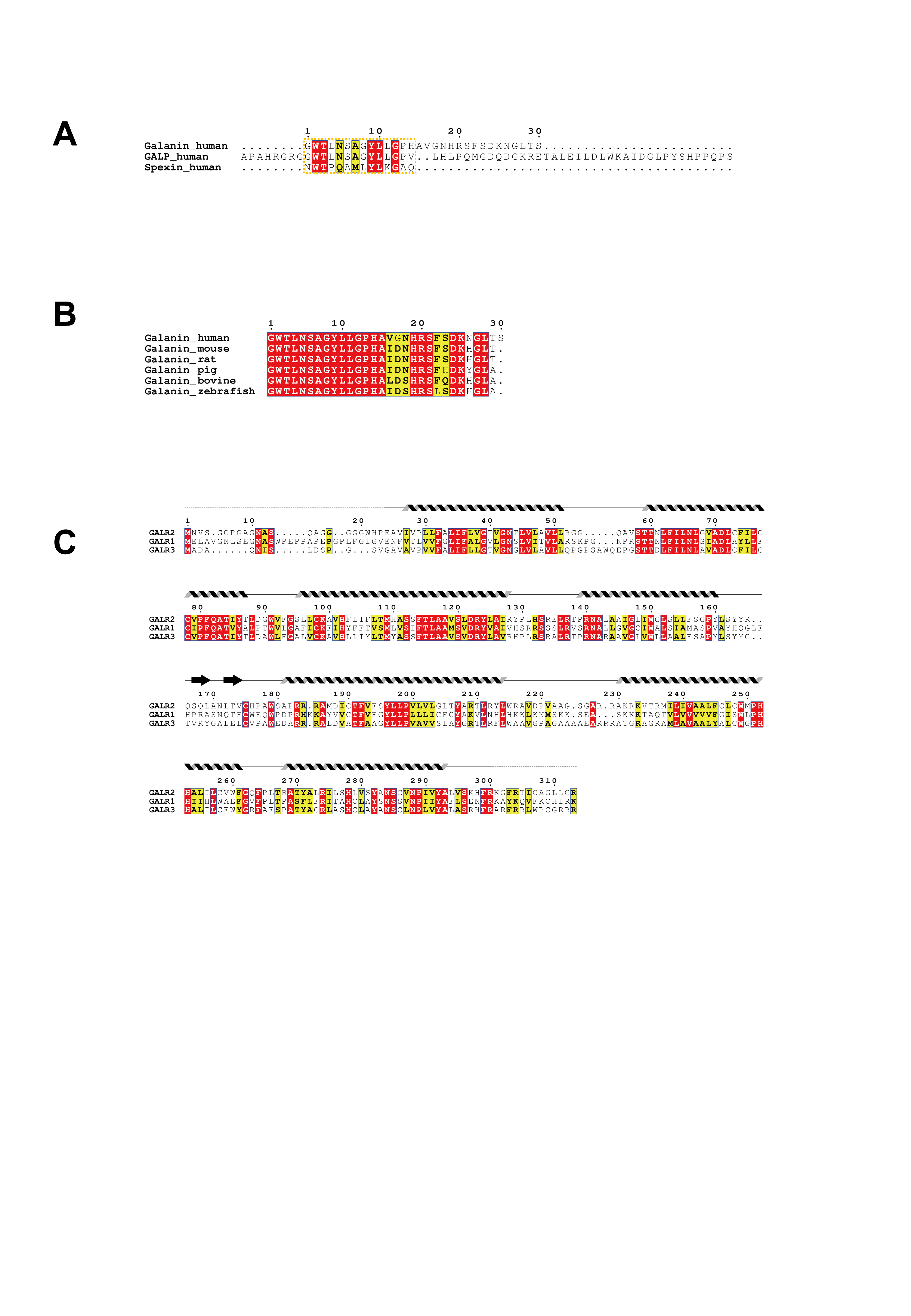

Supplement: S3 Fig — (A) The sequences of human galanin, GALP, and spexin are aligned. Totally conserved residues are indicated by red squares, and similarly conserved residues are indicated by yellow squares. The essential region of the ligands for binding to GALR2 is indicated by an orange dotted square. (B) The sequences of galanin from various species are aligned. Only the galanin from human is composed of 30 residues. (C) The sequences of human GALRs are aligned. The secondary structures of the GALR2 are shown above the sequences. GALP, galanin-like peptide; GALR, galanin receptor. (TIF) [file pbio.3001714.s003.TIF]

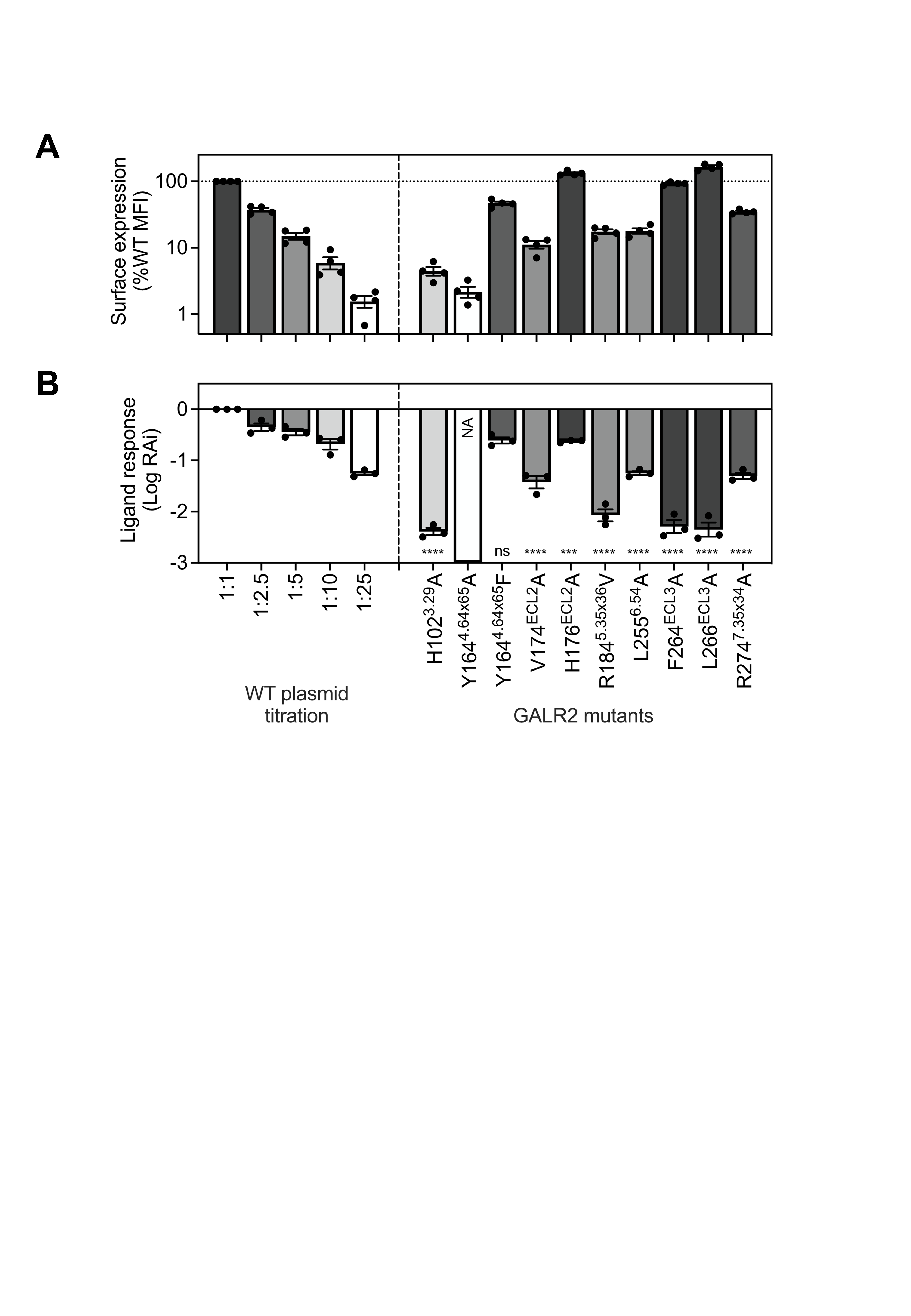

Supplement: S4 Fig — (A) Flow cytometry analysis of WT and mutant GALR2. N-terminally FLAG-epitope-tagged GALR2 constructs were subjected to the flow cytometry using a FLAG-epitope tag antibody. MFI of the mutants was normalized to WT (1:1) after subtracting that of mock. Symbols and error bars indicate mean and SEM, respectively, of 4 independent experiments (dots) with each performed in duplicate. (B) Gq-activity parameters of WT and mutant GALR2. For the individual NanoBiT Gq-PLCβ experiments (Fig 3), RAi of the mutant to that of WT was calculated from Emax and EC50 values, and its logarithm-transformed value (Log RAi) was used to represent Gq-activity parameter. The parameter of Y164A was not available due to the undetectable Gq activity of the mutant. Colors in the mutant bars indicate an expression level matching to that of titrated WT. Bars and error bars represent mean and SEM of 3 independent experiments (dots). NA, parameter not available because of lack of the ligand response. Statistical analyses were performed using the ordinary one-way ANOVA followed by the Dunnett’s post hoc test with the expression-matched (colored) WT response. ns, p > 0.05; ***, p < 0.001; ****, p < 0.0001. The data underlying this figure can be found in S3 Data. MFI, mean fluorescence intensity; RAi, relative intrinsic activity; WT, wild-type. (TIF) [file pbio.3001714.s004.TIF]

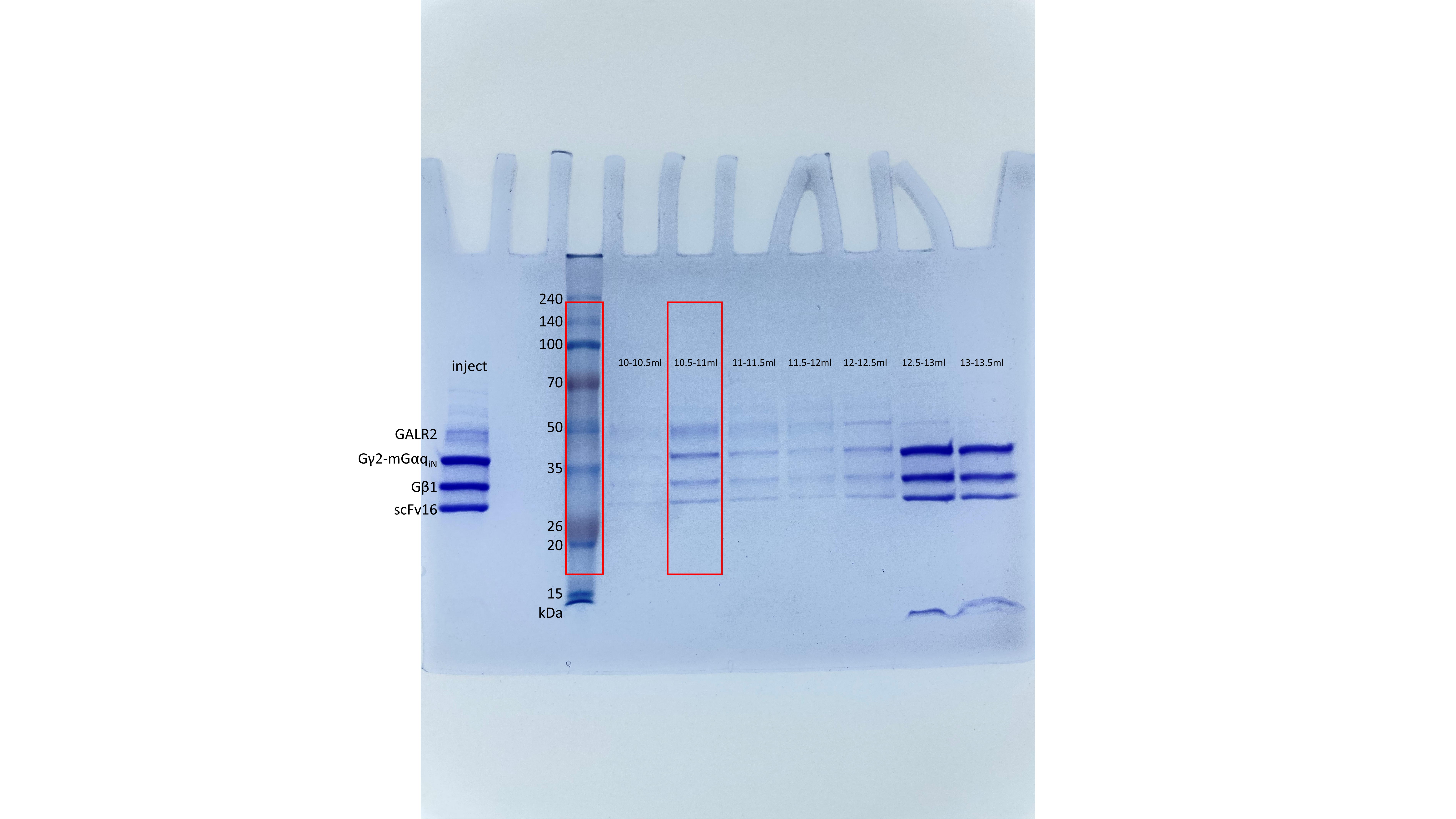

Supplement: S1 Raw Images — The injected and eluted SEC samples were analyzed using SDS-PAGE. The red boxes were used to indicate the cropped parts used in S1 Fig. (TIF) [file pbio.3001714.s008.tif]
